# Prevalence of Skin Reactions and Self-Reported Allergies in 5 Countries with Their Social Impact Measured through Quality of Life Impairment

**DOI:** 10.3390/ijerph18094501

**Published:** 2021-04-23

**Authors:** Samir Salah, Charles Taieb, Anne’ Laure Demessant, Marek Haftek

**Affiliations:** 1 La Roche-Posay Dermatological Laboratories, 92300 Levallois-Perret, France; anne-laure.demessant@loreal.com; 2 European Market Maintenance Assessment, 94120 Fontenay-sous-Bois, France; charles.taieb@emma.clinic; 3 CNRS UMR5305, Tissue Biology and Therapeutic Engineering Laboratory, LBTI, 69367 Lyon, France; marek.haftek@univ-lyon1.fr

**Keywords:** allergies, skin manifestations, quality of life, skin barrier dysfunction, moisturizers

## Abstract

Background: The prevalence of allergies increases worldwide. Allergies may increase the risk of skin reactions. Objective: To evaluate the prevalence of allergies and skin reactions in the adult population, the strength of their relationship, and their impact on the quality of life. Methods: An online survey was conducted in a representative population of 11,067 adults from China, USA, Brazil, Russia, and France. Results: Overall, 35.6% of respondents reported having allergies, they were predominantly fair-skinned women, and younger than responders reporting no allergy. Among patients reporting allergies, 68.6% declared that their allergy makes their skin reacts. A strong association between allergy and major skin reactions was observed, which were associated with skin discomforts such as itching, burning, and pain. Skin discomforts were associated with an increased risk of quality of life alteration. Conclusions: Quantifying the prevalence and the association of allergies with skin reactions and discomfort sensations is critical to evaluating the impact on quality of life. Since skin barrier alteration is hypothesized as a risk factor and a route of sensitization for allergy development, the daily use of topical treatments, such as moisturizers, could help prevent allergic skin reactions, discomfort and impaired quality of life in individuals with an altered skin barrier.

## 1. Introduction

The increasing occurrence of allergies, including food allergy, asthma, allergic rhinitis, has even been referred to as an “allergy epidemic”, which is much observed in regions with rapid economic growth and massive urbanization [1,2,3,4,5].

Allergies may present with varied symptoms, including skin reactions such as urticaria, angioedema, atopic eczema, or contact dermatitis, as well as skin sensations, sometimes independent from the occurrence of skin lesions, such as pruritus, flushing, burning, and pain [6,7,8]. Skin reactions can have a substantial impact on the quality of life (QoL) of patients, which is a subjective status of wellbeing in the emotional, physical, social, and functional dimensions of an individual. The burden of disease, as a patient perceives it, is usually described by patients in terms of symptoms and impact on QoL [9,10,11,12,13]. It is, therefore, crucial to quantify the risk increase of skin reaction incidence in the presence of allergy.

Skin barrier impairment has been proposed as a risk factor and a route of sensitization for the development of food allergy and for the occurrence of allergic rhinitis and of allergic asthma [14,15]. Chemically or physically induced barrier dysfunction can be a cause of irritant or allergic contact dermatitis [16]. Indeed, inborn or acquired defects in skin barrier function, often associated with a chronic inflammatory reaction, may facilitate allergen entry and activation of immune priming, leading to local or systemic allergic responses.

The definition of precise causes of allergies often requires rigorous tests such as oral food challenge or patch testing performed by specialized healthcare professionals. This renders the prevalence of allergy in the general population difficult to estimate. Nevertheless, self-reported allergy prevalence represents an accessible means to assess and understand the burden of allergies. 

So far, little is known about the strength of the relationship between allergy and skin reactions and associated discomfort sensations which ultimately generate a social burden that we can measure through quality of life impairment. Here, the data of an online survey assessing the prevalence of self-reported allergy and skin conditions, in the adult population in five countries: China, USA, Brazil, Russia, and France are reported. 

## 2. Materials and Methods

### 2.1. Study Population

This online survey was conducted between December 2018 and January 2019 by a research house (HC Conseil Paris, Paris, France) using a quota sampling method for each country (sex, age, socio-professional status, and regional distribution). A total sample of 11,067 adult individuals was recruited, constituted of 2036 French, 2008 US Americans, 2010 Russians, 3010 Chinese, and 2003 Brazilians.

### 2.2. Survey

In addition to sociodemographic data, the survey also collected data on skin characteristics, lifestyle, exposome, allergies, medical diagnosis, skin reactions, discomfort sensations, and therapeutic treatments. Unfortunately, the quality of life questionnaire was only administered on a subset of 206 French patients with a persistent cough. Therefore, the impact of skin discomfort in terms of risk increase on quality of life impairment will be evaluated only on this subset. See Supplementary Materials in S1 for questionnaire details. 

### 2.3. Statistical Analysis

Comparison between participants who reported allergies and participants who did not report any allergy can be found in published studies [17,18,19]. Comparisons of incidences between groups through 2 × 2 contingency tables were performed using a Fisher’s exact test. Relative risk (RR) was calculated and displayed as effect size metric. For 3 × 3 and more contingency tables, a Chi-square test was used. The statistical level of significance was set at 5%. Statistical analyses were performed using the Python SciPy package (version 1.5.2., http://www.scipy.org, accessed on 23 April 2021).

## 3. Results

### 3.1. Demographic Description

The characteristics of the populations with no allergy and with allergies are detailed in Table 1.

These demographic details show that compared to those not reporting allergy, respondents who reported suffering from allergies were younger, with an over-representation of women and fair skin phototypes. 

### 3.2. Allergy Population

The estimated prevalence of the different allergies and skin reactions are described in Table 2.

The proportion of allergy-reporting respondents declaring that their allergy makes their skin react was high (68.6%). The incidence of various types of skin reactions in individuals reporting, or not, various types of allergy was evaluated (Table 3). 

A significant increase of all types of skin reactions in the presence of reported allergy was observed. Therefore, the incidence of skin discomforts on respondents with at least one described skin reaction was investigated (Table 4). Pruritus, burning sensations, and pain were significantly associated with skin reactions.

Next, an increased risk of QoL alteration was investigated in patients with or without skin discomforts. The risk of QoL impairment was increased in the presence of skin discomforts like pain and burning. For pruritus, even if the comparisons of some attributes are not statistically significant, the effect size remains relatively high (Table 5).

## 4. Discussion

In this self-reported survey on the representative adult population of five major countries, 35.6% of respondents reported having allergies, with symptoms such as allergic rhinitis, atopic dermatitis, asthma, edema, bronchitis, and conjunctivitis. One can extrapolate from these data that they were predominantly fair-skinned women, younger than those responders reporting no allergy. This proportion is similar to those reported in previous studies [17,21]. In most cases (73.5%), the reported allergies were diagnosed by a physician.

Among individuals reporting allergies, 68.6% declared that their allergy makes their skin react. The prevalence of skin reactions of various types were measured: acne, contact eczema, atopic dermatitis, rosacea, psoriasis, vitiligo, or seborrheic dermatitis. Interestingly, an increase of all types of skin reactions was observed in the presence of reported allergy. These skin reactions were most often associated with skin discomforts such as itching, burning, and pain. Skin discomfort was associated with QoL alterations such as fatigue, embarrassment, anxiety, difficulties with day-to-day management, and altered quality of sleep. 

The role of the skin barrier in allergic sensitization has been well-described [16]. Skin, as the first frontier between the body and the environment, is a potential route of allergen penetration and plays a predominant role in the development of allergies. An intact epidermal barrier protects from exposure to exogenous allergens, whereas physical or functional impairment of the skin barrier promotes sensitization [22]. 

The present study, conducted on a large and ethnically heterogeneous population of industrialized and developing countries, highlights the fact that the perception of skin aggression is generalized. Indeed, even in the absence of diagnosed allergy or allergic symptoms, the likelihood of individuals presenting with “dry”, “sensitive” or “fragile” skin is increasing, and those individuals are prone to experience skin reactions similar to those described in the present report. In this study, the actual presence or absence of existing allergies could not be verified. 

Another aspect that the present study points to is the important role of the skin barrier –reinforcing the importance of topical treatments, capable of improving skin defenses without introducing potentially allergenic components. In this context, the role of the skin microbiota should also be raised. Indeed, the skin is an ecological niche for a wide range of microorganisms, which are, in their majority, harmless or beneficial, providing protection against pathogens and playing a role in modulating the cutaneous innate and adaptive immune systems [23]. Some bacteria can provide protection against inflammation, while others may cause a long-lasting cutaneous inflammation [24,25,26]. 

Dysbiosis, or disruption of balance in the microbiome, has been extensively studied in the context of atopic dermatitis, the first step of the “atopic march”, which can subsequently lead to allergic rhinitis and asthma [27]. Moreover, since the skin microbiota is under the influence of the skin water content [28], the importance of skin moisturizers for the maintenance of the skin barrier and of the well-balanced skin microbiome through a targeted bacteria inhibition and a prebiotic activity has been highlighted [29]. 

The reported presence of allergies and skin reactions with no explicit validation by an independent medical staff may be one limitation of this study. Indeed, survey participants can misinterpret allergy and/or cutaneous symptoms, e.g., an adverse effect of food intake, and without the medically proven evidence of immune mechanism, the self-observed symptoms may easily be mistaken to be an allergic reaction and/or a skin reaction and reported as such [30]. Another limitation of this approach is that only adults of 18 years old and older were sampled, whereas allergy rates are increasing most rapidly among children [5]. Nevertheless, it is hardly conceivable to perform a reliable questionnaire survey via e-mail concerning QoL in toddlers. 

## 5. Conclusions

Quantifying the prevalence and the association of allergies, skin reactions, and discomfort sensations with their ultimate social impact translated from quality of life alterations, is critical for diseases like allergies as they affect a growing population in industrialized and developed countries. Several environmental factors can interfere with the skin barrier function and the skin microbiota. Daily use of topical treatments, such as moisturizers, aiming at the reinforcement of the skin barrier and/or balancing the skin microbiome, should help prevent allergic skin reaction in individuals with an altered skin barrier. Such relief of discomfort feelings could contribute to a better quality of life.

## Figures and Tables

**Table 1:** Characteristics of the populations.

	No Allergy	With Allergies	
**Participants (%)**	7125 (64.4%)	3942 (35.6%)	
**Age**			
Mean (SD)	41.5 (14.7)	39.83 (14.1)	<0.001
**Gender**			
Female	3443 (48.3%)	2142 (54.3%)	<0.001
Male	3682 (51.7%)	1800 (45.7%)	
Skin phototype *			
I	857 (12.0%)	587 (14.9%)	<0.001
II	1875 (26.3%)	1116 (28.3%)	
III	2741 (38.5%)	1331 (33.8%)	
IV	1134 (15.9%)	635 (16.1%)	
V	270 (3.8%)	147 (3.7%)	
VI	248 (3.5%)	126 (3.2%)	

*: skin phototypes according to the Fitzpatrick classification [20].

**Table 2:** Type of allergies and skin reaction potential.

**Responders with** **A** **llergy** **(*n*):**	3942
Diagnosed allergy *	2897 (73.5%)
**Who diagnosed the allergy**	
Dermatologist	1191 (41.1%)
General practitioner	830 (28.7%)
Allergy specialist	608 (21.0%)
ENT doctor	98 (3.4%)
Pediatrician	61 (2.1%)
Pulmonary specialist	51 (1.8%)
Other specialized physician	39 (1.3%)
Homeopathic doctor	14 (0.5%)
Acupuncturist	5 (0.2%)
**Type of allergy reported:**	
Respiratory	2247 (57.0%)
Skin	2513 (63.7%)
Food	1764 (44.7%)
**Allergy symptoms**	
Allergic rhinitis	1870 (47.4%)
Atopic dermatitis	1213 (30.8%)
Asthma	481 (12.2%)
Edema	444 (11.3%)
Bronchitis	406 (10.3%)
Conjunctivitis	379 (9.6%)
**Type of allergens**	
Pollens	1742 (44.2%)
Dust mites	1363 (34.6%)
Mold	1080 (27.4%)
Food	1076 (27.3%)
Animals	769 (19.5%)
Cockroaches	309 (7.8%)
Latex	287 (7.3%)
Hymenoptera	254 (6.4%)
**Participants reporting that their allergy make their skin react:**	
	2706 (68.6%)

**Table 3:** Allergy and skin reactions.

	No Diagnosed Allergy	Diagnosed Allergy	RR (*p*-Value)
Acne	1664 (21.0%)	919 (32.4%)	1.54 (<0.001)
Contact eczema	498 (6.4%)	641 (23.5%)	3.68 (<0.001)
Atopic dermatitis	710 (9.2%)	824 (30.4%)	3.3 (<0.001)
Rosacea	520 (6.7%)	450 (16.5%)	2.45 (<0.001)
Psoriasis	413 (5.3%)	329 (11.8%)	2.25 (<0.001)
Vitiligo	133 (1.7%)	156 (5.7%)	3.3 (<0.001)
Seborrheic dermatitis	697 (9.0%)	582 (21.5%)	2.38 (<0.001)

**Table 4:** Skin reactions and discomfort sensations.

	No Skin Reaction 6039 (54.6%)	Skin Reactions 5028 (45.4%)	RR (*p*-Value)
Itching	1789 (29.6%)	2793 (55.5%)	1.88 (<0.001)
Burning	1043 (17.3%)	1700 (33.8%)	1.96 (<0.001)
Pain	1123 (18.6%)	1609 (32.0%)	1.72 (<0.001)

**Table 5:** Skin discomfort and impact on the quality of life.

	With Skin Discomfort *n* (%)	Without Skin Discomfort *n* (%)	RR (*p*-Value)
**Pain (*n*)**	138	68	
Fatigue	30 (21.7%)	28 (41.2%)	1.89 (0.005)
Embarrassment	48 (34.8%)	35 (51.5%)	1.48 (0.024)
Anxiety	25 (18.1%)	28 (41.2%)	2.27 (<0.001)
Difficulties with day-to-day management	13 (9.4%)	23 (33.8%)	3.59 (<0.001)
Altered quality of sleep	25 (18.1%)	30 (44.1%)	2.44 (<0.001)
**Burning (*n*)**	66	140	
Fatigue	29 (20.7%)	29 (43.9%)	2.12 (<0.001)
Embarrassment	43 (30.7%)	40 (60.6%)	1.97 (<0.001)
Anxiety	24 (17.1%)	29 (43.9%)	2.56 (<0.001)
Difficulties with day-to-day management	13 (9.3%)	23 (34.8%)	3.75 (<0.001)
Altered quality of sleep	22 (15.7%)	33 (50.0%)	3.18 (<0.001)
**Itching (*n*)**	104	102	
Fatigue	24 (23.1%)	34 (33.3%)	1.44 (0.122)
Embarrassment	35 (33.7%)	48 (47.1%)	1.4 (0.065)
Anxiety	18 (17.3%)	35 (34.3%)	1.98 (0.007)
Difficulties with day-to-day management	11 (10.6%)	25 (24.5%)	2.32 (0.01)
Altered quality of sleep	18 (17.3%)	37 (36.3%)	2.1 (0.003)

## Supplementary Materials

The following are available online at https://www.mdpi.com/article/10.3390/ijerph18094501/s1, S1: Subset of the survey questionnaire.
