# Supplementary material for: Prevalence of Skin Reactions and Self-Reported Allergies in 5 Countries with Their Social Impact Measured through Quality of Life Impairment
Source: Int J Environ Res Public Health. 2021 Apr 23;18(9):4501. doi: 10.3390/ijerph18094501 (PMC8123031; doi:10.3390/ijerph18094501)
Supplement: Supplementary file 1 [file ijerph-18-04501-s001.zip › ijerph-1120285-supplementary.pdf]

Subset of the survey questionnaire

[S1]: You are:

1 A man
2 A woman

[S2]: What is your age?

[Q1]: Among the following phototypes, specify the one to which you feel closest?

1	You have very white skin, blonde or red hair, and blue / green eyes with often freckles. Sunburns are systematic, your skin never tans and always reddens.
2	You have fair skin, red to brown blonde hair, and light to brown eyes with occasional freckles. Sunburns are common, your skin barely tans or very slowly
3	You have medium skin tone, brown hair, and brown eyes. Sunburns are occasional and your skin gradually tans.
4	You have dark skin tone and brown / black hair and eyes. Sunburn is occasional, especially during intense exposure. Your skin tans well.
5	You have dark brown skin, black hair and eyes. Sunburns are rare, your skin tans a lot.
6	You have black skin, black hair and eyes. Sunburns are very exceptional

[Q5]: When you were a child, did you have eczema or atopic dermatitis?

1 Yes
2 No
3 Do not know

[Q6]: Do you currently consider your skin?

1 Dry
2 Normal

3	Greasy
4	Mixt

[Q9]: Do you currently consider your skin?

1	Very sensitive
2	Quite sensitive
3	A little sensitive
4	Not sensitive
5	I couldn't say

Q10: Currently, do you experience on your skin, at least once a day, one or more of the following sensations?

	0	No feeling
…		
	100	Maximum felt imaginable
[Q10r1]		Burning
[Q10r2]		tightness
[Q10r3]		Pain
[Q10r4]		Tickling
[Q10r5]		Numbness
[Q10r6]		Hot flushes
[Q10r7]		Pruritus

*: a presence/absence of a discomfort is defined as a value above 35

Q11: Could you please clarify if you are currently suffering from…?

	1	Yes
	2	No
	3	Do not know
[Q11r1]		Acne
[Q11r2]		Contact eczema
[Q11r3]		Atopic dermatitis
[Q11r4]		Rosacea
[Q11r5]		Psoriasis

[Q11r6]	Vitiligo
[Q11r7]	Seborrheic dermatitis
[Q11r8]	A scalp problem or disease
[Q11r9]	Sun allergy
[Q11r10]	Other skin disorder

[Q51]: Do you have any allergies?

1	Yes
2	No

[Q55]: Does your allergy cause your skin to react?

1	Yes
2	No

Q52_followup: Do you have or have you had...

1	Yes
2	No
[Q52_followupr1]	a food allergy?
[Q52_followupr2]	a respiratory allergy (rhinitis or asthma)?
[Q52_followupr3]	a skin allergy?

[Q56]: Has a doctor diagnosed your allergy?

1	Yes
2	No

[Q56_1]: was it a...

1	general practitioner
2	dermatologist
3	allergy specialist
4	pulmonary specialist
5	ENT doctor
6	pediatrician

C1 - Internal use

7	acupuncturist
8	homeopathic doctor
9	other specialized physician

[Q57]: Do you know what your allergy is called?

1	Yes
2	No

Q58: What are the symptoms of your allergy?

	0	Unchecked
	1	Checked
[Q58r1]		asthma
[Q58r2]		allergic rhinitis (hay fever)
[Q58r3]		bronchitis with wheezing
[Q58r4]		eczema/atopic dermatitis
[Q58r5]		conjunctivitis
[Q58r6]		edema
[Q58r7]		other

Q59: Do you know the allergen?

	0	Unchecked
	1	Checked
[Q59r1]		dust mites
[Q59r2]		cockroaches
[Q59r3]		pollens
[Q59r4]		food allergens
[Q59r5]		dogs, cats, ferrets, other animals
[Q59r6]		mold
[Q59r7]		Hymenoptera
[Q59r8]		latex
[Q59r9]		other
[Q59r10]		Je ne sais pas ce qui déclenche mon allergie

C1 - Internal use

[Q64]: Do you have a cough that has lasted for more than 2 weeks?

1 Yes
2 No

[Q70_2r1]: In the past 2 weeks, my cough makes me feel tired:

7 All the time
6 Most of the time
5 A good part of the time
4 Sometimes
3 Few times
2 Almost never
1 Never

*: a presence of a quality of life impact is defined as an answer to one of the three values ("All the time", "Most of the time", "A good part of the time")

[Q70_3r1]: During the past 2 weeks, I feel embarrassed about my cough:

7 All the time
6 Most of the time
5 A good part of the time
4 Sometimes
3 Few times
2 Almost never
1 Never

*: a presence of a quality of life impact is defined as an answer to one of the three values ("All the time", "Most of the time", "A good part of the time")

[Q70_4r1]: During the past 2 weeks I have felt anxious about my cough:

7 All the time
6 Most of the time
5 A good part of the time
4 Sometimes
3 Few times

C1 - Internal use

2 Almost never
1 Never

*: a presence of a quality of life impact is defined as an answer to one of the three values ("All the time", "Most of the time", "A good part of the time")

[Q70_5r1]: During the past 2 weeks, my cough has interfered with the smooth running of my work or other daily tasks:

7 All the time
6 Most of the time
5 A good part of the time
4 Sometimes
3 Few times
2 Almost never
1 Never

*: a presence of a quality of life impact is defined as an answer to one of the three values ("All the time", "Most of the time", "A good part of the time")

[Q70_6r1]: Over the past 2 weeks, I have felt that my cough is preventing me from enjoying my life to the fullest:

7 All the time
6 Most of the time
5 A good part of the time
4 Sometimes
3 Few times
2 Almost never
1 Never

*: a presence of a quality of life impact is defined as an answer to one of the three values ("All the time", "Most of the time", "A good part of the time")

[Q70_7r1]: During the past 2 weeks, my cough interfered with my sleep:

7 All the time
6 Most of the time
5 A good part of the time
4 Sometimes
3 Few times

C1 - Internal use

2	Almost never
1	Never

*: a presence of a quality of life impact is defined as an answer to one of the three values ("All the time", "Most of the time", "A good part of the time")

C1 - Internal use
